# Influence of Gamma Radiation on the Damping Property of Magnetorheological Elastomer

**DOI:** 10.3390/polym14183708

**Published:** 2022-09-06

**Authors:** Guojiang Liao, Wenzheng Zhang, Qingna Zeng, Xiangfeng Peng, Wanjun Wu, Shuai Liu, Bin Lan, Yixiong Zhang

**Affiliations:** Science and Technology on Reactor System Design Technology Laboratory, Nuclear Power Institute of China, Chengdu 610213, China

**Keywords:** magnetorheological elastomer, gamma radiation, damping, mechanical property

## Abstract

Magnetorheological elastomer (MRE) is a kind of smart material, whose mechanical property can be controlled by the external magnetic field quickly and reversibly. The damping property of MRE is one of the most concerned properties when designing MRE based devices. In this work, the influence of gamma radiation on the damping property of MRE was investigated. Six different exposures of gamma radiation were applied to the MRE samples. The highest gamma radiation dose was up to 1 × 10^5^ Gy(Si), which can cover most of the engineering application scenarios. The influence of gamma radiation on the damping-strain relation and the damping-magnetic-field relation were studied. The probable mechanisms were discussed in detail. It is found that the gamma radiation does not affect the variation trend of loss factor of MRE with increasing strain amplitude or magnetic flux density. But it affects the variation trend of the maximum change of strain-induced or magnetic-field-induced loss factor of MRE. Besides, with constant strain and constant magnetic flux density, the loss factor of MRE shows w-shape variation trend with increasing gamma radiation dose. It is considered to be resulted from the combined action of the intrinsic damping and the interfacial friction damping of MRE.

## 1. Introduction

Magnetorheological elastomer (MRE) is considered as a kind of smart material with controllable properties [1,2]. Such material is usually composed of micro-sized ferromagnetic particles dispersed into a non-magnetic polymeric matrix and other additives [2,3]. When applied an external magnetic field, its properties, such as storage modulus, damping ratio, electrical impedance, tribological characteristics, surface morphology and so on, can be controlled rapidly and reversibly due to the magnetic interactions among the ferromagnetic particles [1,4,5]. Due to these unique controllable properties by the external magnetic field, MRE has attracted much attention in the engineering applications, such as vibration absorber [6,7,8,9], vibration isolator [10,11,12], sensor [13,14,15], flexible gripper [16] and so on.

The damping property is one of the most concerned properties for MRE. It is important for the performance of some MRE based devices, such as vibration absorber and vibration isolator [10,17]. Therefore, much attention has been paid to the damping property of MRE and the corresponding mechanism [5,17,18,19,20,21,22,23,24,25,26,27,28]. Demchuk investigated the influence of particle size on the damping ratio of MRE. It is found that the damping ratio of MRE decreased with increasing particle size [25]. Chen investigated the effects of matrix type, strain amplitude, excitation frequency and particle content on the damping ratio of MRE [24]. Sun studied the damping property of isotropic MRE and aligned MRE [23]. Fan investigated the influence of interfacial adhesion, interfacial friction and crosslink density on the damping property of MRE [17,21,22]. Tong reported the damping property of MRE with flower-like cobalt particles [5]. Based on the experimental results, Deng and Yang proposed interfacial models to characterize the damping property of MRE referring to the Eshelby and mixed rate criteria [26,28]. These works lay the foundation for investigating the damping property of MRE and make us a relatively deep understanding of the damping property of MRE. However, more work needs to be done to investigate the damping property of MRE, when considering the application of MRE in various engineering environments.

In recent years, MRE has attracted more and more attention in the engineering applications. When used in engineering application, MRE may be exposed to various environments, such as high temperature, humidity, vibration, radiation and so on. It is necessary to investigate the property of MRE under these environments, which is helpful to the design of MRE based devices [29,30,31]. Among the various environments, gamma radiation is one of the concerned, which is common in the applications of nuclear power station and aerospace. MRE is ferromagnetic particle reinforced composite material. Its properties are decided by the particle, matrix and the particle/matrix interface. When exposed to gamma radiation, the ferromagnetic particle, polymeric matrix and the particle/matrix interface would be influenced due to the radiation-induced crosslinking, radiation-induced degradation and modification of the polymer/filler interface [30,31,32,33,34,35]. Considering the importance of the damping property of MRE in the engineering application, it is necessary to investigate the damping property of MRE under gamma radiation. Understanding the corresponding mechanism would be much helpful to the design of MRE based devices in the environment containing gamma radiation.

In this work, the influence of gamma radiation on the damping property of MRE is studied by experiment. The MRE samples were first fabricated by using nature rubber as the matrix. Then, the MRE samples were exposed to six different gamma radiation doses. The highest gamma radiation dose was up to 1 × 10^5^ Gy(Si), which can cover most of the engineering application scenarios. The damping properties of MRE with different gamma radiation exposures were evaluated by using rheometer. The influences of gamma radiation on the damping-strain relation and the damping-magnetic-field relation were investigated. The results in this work would be helpful in the fabrication of MRE material and the design of MRE based device when considering the gamma radiation environment.

## 2. Experimental Details

### 2.1. Sample Preparation

**Figure 1:**
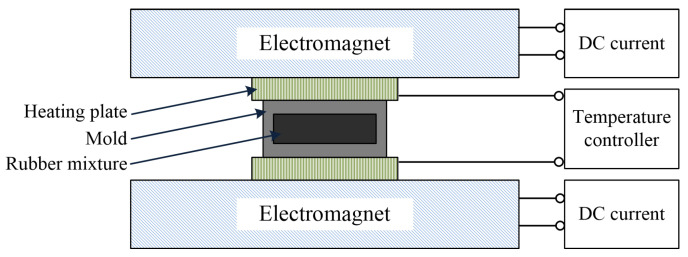
Sketch of the customized magnet-heat device.

The materials of fabricated MRE samples consisted of nature rubber, carbonyl iron particles, plasticizer and other additives. The type of the carbonyl iron particles was CN with the average diameter of 6 μm, from BASF Co. in Heidelberg, Germany. The plasticizer and the other additives were from Hefei Wangyou Rubber Company of China, (Hefei, China). The procedures of fabricating MRE included three steps: mixing, pre-forming configuration and curing. During fabrication, all the raw materials were firstly mixed homogeneously using a double-roll mill (Taihu rubber Machinery Inc., Wuxi, China, Model XK-160). Then the resulting material was compressed into a mold. The material with the mold was fixed to a customized magnet-heat device, whose sketch is shown in Figure 1. This device can keep the rubber mixture at a fixed temperature and supply a vertical magnetic field. The temperature in this step was 120 °C and the magnetic field was roughly 2 T. In this step, the carbonyl iron particles were aligned along the direction of the magnetic field and formed chain-like structures. 30 min later, the procedures of pre-forming configuration finished. Finally, the temperature was raised up to 160 °C for vulcanizing. The procedure of curing lasted for 15 min. After that, the MRE sample was obtained. The mass ratio of the carbonyl iron particles in the prepared MRE samples was 80%. The dimensions of the sample were 20 mm in diameter and 1 mm in thickness.

Beside the MRE samples, the pure nature rubber, i.e., the matrix of MRE, was also fabricated. The raw materials of the pure nature rubber were the same as the MRE except the carbonyl iron particles. The procedures of fabricating pure nature rubber were also the same as the MRE samples. Each sample of the pure nature rubber was 20 mm in diameter and 1 mm in thickness.

### 2.2. Radiation Exposure

The as-prepared MRE samples, the nature rubber and the carbonyl iron particles were exposed to gamma radiation from a Co-60 source at room temperature and in air atmosphere. The average dose rate of gamma radiation was 5000 Gy(Si)/h. Six different gamma radiation doses, including 0 Gy(Si), 2 × 10^4^ Gy(Si), 4 × 10^4^ Gy(Si), 6 × 10^4^ Gy(Si), 8 × 10^4^ Gy(Si) and 1 × 10^5^ Gy(Si), were applied to the samples. After gamma exposure, the samples were kept at room temperature and in air atmosphere for at least 24 h. After no radiation can be detected from the samples, the subsequent characterization for the samples can be carried out.

### 2.3. Characterization

The loss factor and storage modulus of the MRE samples and pure nature rubber matrix before and after gamma radiation exposure were measured by using a plate-plate magneto-rheometer (Physica MCR301, Anton Paar, Graz, Austria) in shear oscillatory mode with different strain amplitudes and magnetic flux densities. The magnetic current varied from 0 A to 5 A, which represented the magnetic flux density applied to the samples varied from 0 mT to 1130 mT. The shear strain amplitude varied from 0.001% to 1%.

The M-H curve of the carbonyl iron particles was measured by HyMDC (Hysteresis Measurement of Soft and Hard Magnetic Materials) from Metis Instruments & Equipment NV, Leuven, Belgium.

The microstructures of the samples were observed by scanning electron microscope (SEM). A beam voltage of 20.0 kV and beam current of 5.5 nA were used to observe the particles and the particle/matrix interface of MRE samples with the secondary electrons as the imaging signal. In order to limit the charge phenomena due to the low conductivity, the MRE samples and the pure nature rubber samples were cut into flakes and coated with a thin layer of gold prior to SEM observation using ion sputtering.

## 3. Results and Discussion

### 3.1. Influence on the Damping-Strain Relation

**Figure 2:**
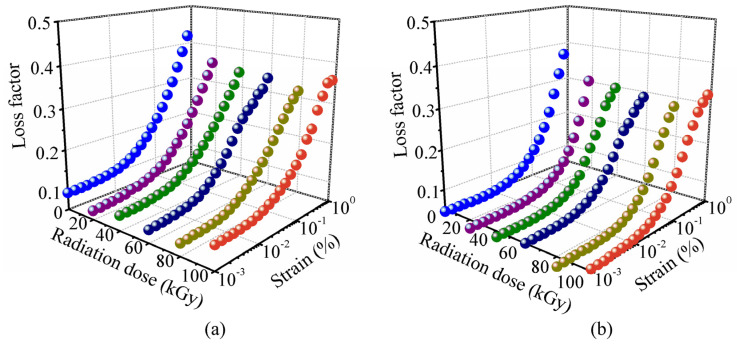
Loss factor of MRE with different strain amplitudes under the external magnetic flux density of (**a**) 0 mT and (**b**) 452 mT.

The damping property of MRE is represented by the loss factor which is the ratio of the loss modulus to the storage modulus. It represents the ability of dissipating energy under external excitation. Figure 2 shows the loss factor of MRE with different gamma radiation exposures at different strain amplitudes. Figure 2a shows the results measured with the external magnetic flux density of 0 mT, i.e., the magnetic current is 0 A for the plate-plate magneto-rheometer. Figure 2b shows the results measured with the external magnetic flux density of 452 mT, i.e., the magnetic current is 2 A for the plate-plate magneto-rheometer. The results measured with the external magnetic flux densities of 226 mT, 678 mT, 904 mT, 1130 mT (1 A, 3 A, 4 A, 5 A for the plate-plate magneto-rheometer respectively) are similar and not shown here. From Figure 2, it can be seen that the loss factor of MRE increases with increasing strain amplitude. This phenomenon is thought to be caused by the intrinsic damping and the interfacial friction damping of MRE [17,28]. The intrinsic damping of MRE mainly comes from the friction among the molecular chains of the rubber matrix. The interfacial friction damping comes from the interfacial friction between the ferromagnetic particles and the matrix. When the strain amplitude increases, the frictions in the particle/matrix interface and among the molecular chains of the rubber matrix become sever. And thus the loss factor of MRE increases with increasing strain amplitude.

**Figure 3:**
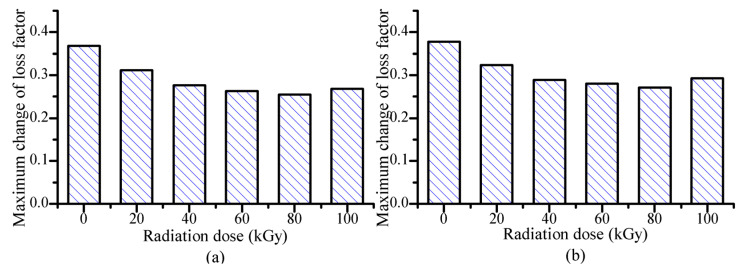
Strain-amplitude-induced maximum change of loss factor of MRE with different gamma radiation exposures (**a**) 0 mT and (**b**) 452 mT.

From Figure 2, the increasing trends of loss factor with increasing strain amplitude are similar for the MRE samples with different gamma radiation exposures. The gamma radiation does not change the variation trend of loss factor with increasing strain amplitude. But it affects the maximum change of loss factor of MRE. Figure 3 shows the maximum change of loss factor with different gamma radiation exposures. Here, the maximum change of loss factor means the difference between the loss factor at strain amplitude of 1% and the loss factor at strain amplitude of 0.001%. It can be seen the maximum change of loss factor decreases with increasing gamma radiation dose. When the radiation dose is higher than 80 kGy(Si), the maximum change of loss factor increases with increasing gamma radiation dose.

**Figure 4:**
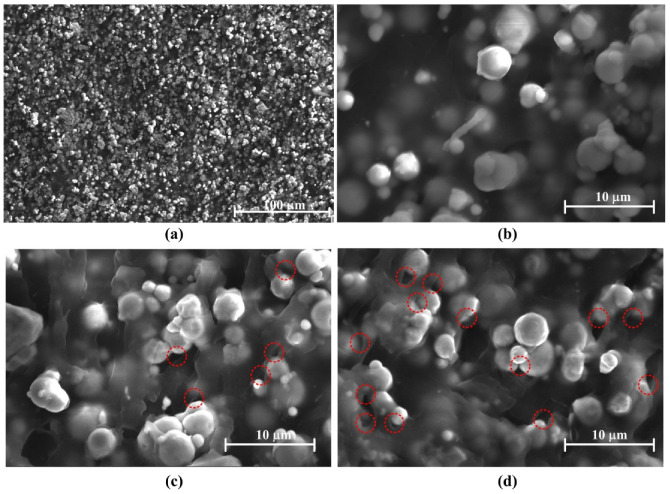
SEM images of MRE with different gamma radiation exposures: (**a**) 40 kGy(Si) (**b**) 40 kGy(Si), (**c**) 80 kGy(Si), (**d**) 100kGy(Si).

When MRE samples are exposed to gamma radiation, the rubber matrix and the particle/matrix interface may change due to the radiation-induced crosslinking and degradation [30,31,32,33,34,35]. When the gamma radiation dose is not high enough, the effect of radiation-induced crosslinking is more obvious than that of radiation-induced degradation. In this case, the particle/matrix interface becomes more compact with increasing gamma radiation dose. The relative motion between the particles and the matrix become difficult, which leads that the interfacial friction in the particle/matrix interface decreases with increasing gamma radiation dose. So, the maximum change of loss factor decreases with increasing radiation dose. When the gamma radiation dose is high enough, the effect of radiation-induced degradation is more obvious than that of radiation-induced crosslinking. In this case, the particle/matrix interface becomes less compact with increasing gamma radiation dose. The relative motion between the particles and the matrix becomes easy, which leads that the interfacial friction increases with increasing gamma radiation dose. So, the maximum change of loss factor increase with increasing gamma radiation dose. Figure 4 shows the microstructures of MRE measured by scanning electron microscopy. Figure 4a,b show the microstructures of MRE with the gamma radiation exposure of 40 kGy(Si). Figure 4c,d show the microstructures of MRE with the gamma radiation exposures of 80 kGy(Si) and 100 kGy(Si) respectively. The microstructures of MRE with the gamma radiation exposures of 0 kGy(Si), 20 kGy(Si) and 60 kGy(Si) are similar to the microstructure of MRE with the gamma radiation exposure of 40 kGy(Si) and are not shown in Figure 4. From Figure 4, the particle/matrix interface is compact when the gamma radiation dose is 40 kGy(Si). When the gamma radiation dose is 80 kGy(Si), some defects can be seen between the particle and the matrix. When the gamma radiation reaches 100 kGy(Si), more defects can be seen. The results from Figure 4 show that the particle/matrix interface become less compact with increasing gamma radiation dose when the gamma radiation dose is higher than 80 kGy(Si). Though the SEM images cannot directly prove that the particle/matrix interface becomes more compact with increasing gamma radiation dose when the radiation dose in lower than 80 kGy(Si), the radiation-induced crosslinking is the main effect when the radiation dose is low. Besides, Figure 4b shows that the particle/matrix interface is compact when the gamma radiation dose is 40 kGy(Si). So, the SEM images can indirectly prove that the particle/matrix interface becomes more compact with increasing gamma radiation dose when the gamma radiation dose is lower than 80 kGy(Si).

Considering the results from Figure 3 and Figure 4 together, it can be concluded that the gamma radiation-induced change of particle/matrix interface leads to the change of maximum change of loss factor of MRE. When the gamma radiation dose is lower than 80 kGy(Si), the gamma radiation makes the particle/matrix interface more compact with increasing radiation dose, which leads the maximum change of loss factor decrease with increasing strain amplitude. When the gamma radiation dose is higher than 80 kGy(Si), the gamma radiation makes the particle/matrix interface less compact with increasing radiation dose, which leads the maximum change of loss factor increases with increasing strain amplitude.

### 3.2. Influence on the Damping-Magnetic-Field Relation

**Figure 5:**
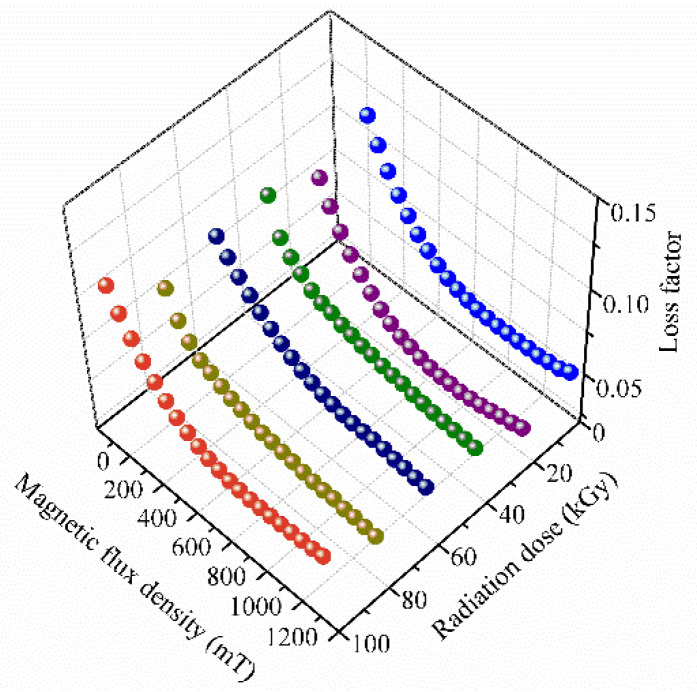
Loss factor of MRE with different magnetic flux densities.

The loss factor of MRE with different magnetic flux densities is shown in Figure 5. It can be seen that the loss factor of MRE decreases with increasing magnetic flux density. This phenomenon is thought to be caused by the variation of the intrinsic damping of the rubber matrix of MRE [17,22]. The intrinsic damping of MRE mainly comes from the friction among the molecular chains of the rubber matrix. For MRE, the ferromagnetic particles are distributed in the rubber matrix. These particles would restrict the motion of the molecular chains, which leads to the decreasing of the intrinsic damping of the rubber matrix. When increasing external magnetic flux density, the interactions among the ferromagnetic particles increase, which would further strengthen the restriction effect of the ferromagnetic particles to the molecular chains of the rubber matrix. And thus, the loss factor of MRE decreases with increasing magnetic flux density.

**Figure 6:**
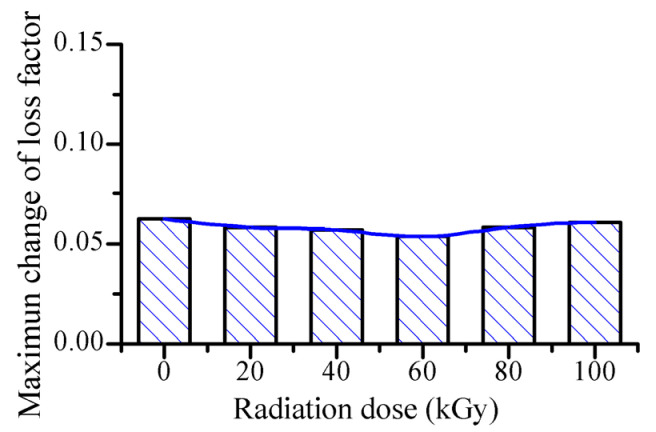
Magnetic-field-induced maximum change of loss factor of MRE with different gamma radiation exposures.

**Figure 7:**
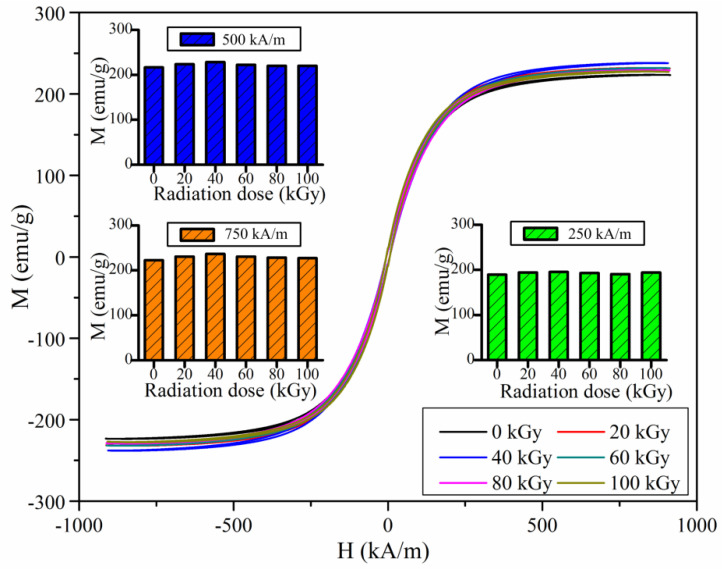
M-H curves of the ferromagnetic particles with different gamma radiation exposures.

From Figure 5, the decreasing trends of loss factor with increasing magnetic flux density are similar for the MRE samples with different gamma radiation exposures. The gamma radiation does not change the variation trend of loss factor with increasing magnetic flux density. Figure 6 shows the maximum change of loss factor with different gamma radiation exposures. Here, the maximum change of loss factor represents the difference between the loss factor with the external magnetic flux density of 0 mT and the loss factor with the external magnetic flux density of 1130 mT. It can be seen that the maximum change of loss factor varies slightly with increasing gamma radiation dose. It varies in the range of 0.054 and 0.062. This slight variation of the maximum change of loss factor may be resulted from the variation of the magnetic property of the ferromagnetic particles. Figure 7 shows the experimental M-H curves of the ferromagnetic particles with different gamma radiation exposures. It can be seen that the magnetization intensity of the ferromagnetic particle varies slightly with increasing gamma radiation dose. So the magnetic interaction among the ferromagnetic particles varies slightly with increasing gamma radiation dose. And thus, the restriction applied to the molecular chains of the rubber matrix by the ferromagnetic particles varies slightly, which leads to the slight variation of the maximum change of the loss factor of MRE with increasing gamma radiation dose.

### 3.3. Influence on the Damping Propertywith Constant Strain and Constant Magnetic Field

**Figure 8:**
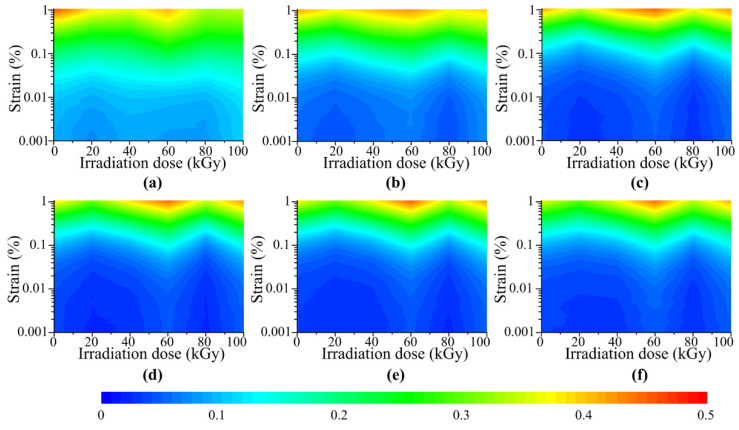
Loss factor of MRE with different radiation doses, strain amplitudes and magnetic flux densities: (**a**) 0 mT, (**b**) 226 mT, (**c**) 452 mT, (**d**) 678 mT, (**e**) 904 mT, (**f**) 1130 mT.

Figure 8 shows the loss factor of MRE with different gamma radiation doses, strain amplitudes and magnetic flux densities. The strain amplitude varies from 0.001% to 1% with 100 data points distributed logarithmically. The gamma radiation dose varies from 0 Gy(Si) to 1 × 10^5^ Gy(Si) with 2 × 10^4^ Gy(Si) as the interval. The magnetic flux density varies from 0 mT to 1131 mT and the corresponding magnetic current of the magneto-rheometer varies from 0 A to 5 A with 1 A as the interval. From Figure 8, an interesting variation trend of loss factor of MRE with increasing gamma radiation dose can be observed. The loss factor of MRE decreases first and then increases when the gamma radiation dose is lower than 6 × 10^4^ Gy(Si). When the gamma radiation dose is higher than 6 × 10^4^ Gy(Si), the loss factor of MRE decreases again and then increases. The loss factor of MRE shows a w-shape variation trend with increasing gamma radiation. From Figure 8, it can also be seen that the variation trends of loss factor of MRE with different magnetic flux densities and strain amplitudes are similar. They all show w-shape variation trends with increasing gamma radiation dose.

**Figure 9:**
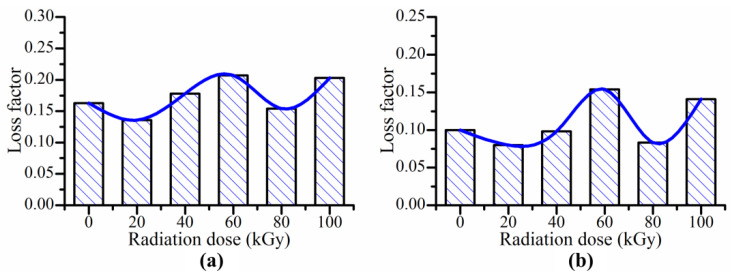
Loss factor of MRE with the strain amplitude of 0.1% and with the magnetic flux densities of (**a**) 226 mT and (**b**) 1131 mT.

To show the variation trend of loss factor of MRE with increasing gamma radiation dose more clearly, the data measured with the magnetic flux densities of 226 mT and 1131 mT and the strain amplitude of 0.1% are picked from Figure 8 and shown in Figure 9. An obvious w-shape variation trend of loss factor of MRE with increasing gamma radiation dose can be seen from Figure 9. The radiation doses corresponding to the peak values of loss factor of MRE are 0 Gy(Si), 6 × 10^4^ Gy(Si) and 1 × 10^5^ Gy(Si) respectively. While the radiation doses corresponding to the valley values of loss factor of MRE are 2 × 10^4^ Gy(Si) and 8 × 10^4^ Gy(Si) respectively. This w-shape variation trend of loss factor of MRE with increasing gamma radiation dose has never been reported. And the damping property of MRE is quite important for the MRE based devices. So, it is necessary to understand this damping behavior MRE.

The damping of MRE consists of intrinsic damping and interfacial friction damping. The intrinsic damping mainly comes from the rubber matrix and the ferromagnetic particles and can be expressed as [17,26,28]

D_i_ = Φ_m_D_m_ + Φ_p_D_p_ (1)

where D_i_, D_m_ and D_p_ are the intrinsic damping of MRE, the intrinsic damping of the rubber matrix and the intrinsic damping of the ferromagnetic particles respectively; Φ_m_ and Φ_p_ are the volume fraction of the rubber matrix and the ferromagnetic particles respectively. Generally, the intrinsic damping of the ferromagnetic particles is much smaller than that of the rubber matrix. So, the intrinsic damping of the MRE is simplified as

D_i_ = Φ_m_D_m_ = (1 − Φ_p_)D_m_ (2)

**Figure 10:**
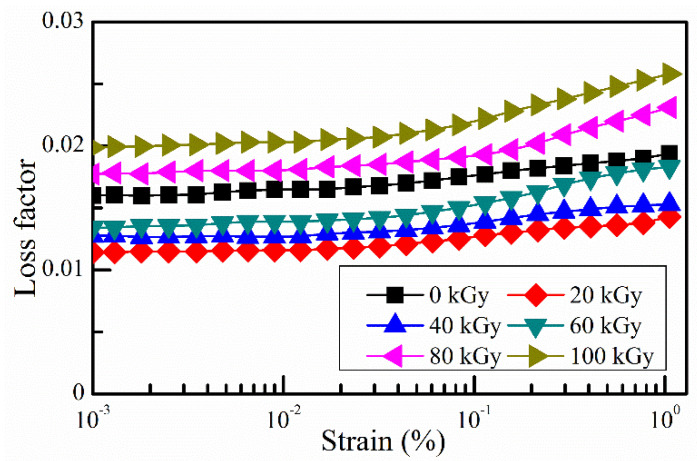
Loss factor of the rubber matrix with different strain amplitudes and different gamma radiation doses.

The intrinsic damping of the rubber matrix mainly comes from the friction among the molecular chains of the rubber matrix. With gamma radiation exposure, the radiation-induced crosslinking and degradation would influence the of relative motion of the molecular chains of the rubber matrix [30,31]. When the gamma radiation dose is not high enough, the radiation-induced crosslinking is more obvious than the radiation-induced degradation. In this case, the molecular chains of the rubber matrix are harder to move with increasing gamma radiation. And thus, the intrinsic damping of the rubber matrix decreases with increasing gamma radiation. When the gamma radiation is high enough, the radiation-induced degradation is more obvious than the radiation-induced crosslinking. In this case, the molecular chains of the rubber matrix are easier to move with increasing gamma radiation dose. And thus, the intrinsic damping of the rubber matrix increases with increasing gamma radiation dose. Therefore, the intrinsic damping of the rubber matrix should decrease first and then increase with increasing gamma radiation dose. Figure 10 shows the experimental result of the loss factor of the rubber matrix with different gamma radiation doses and different strain amplitudes. The experimental result agrees with the analysis. And it indicates that turning point is around 2 × 10^4^ Gy(Si), which means the intrinsic damping of the rubber matrix decreases first with increasing gamma radiation dose and then increases with increasing gamma radiation dose when the gamma radiation dose exceeds 2 × 10^4^ Gy(Si). The intrinsic damping shows a V-shape variation trend with increasing gamma radiation dose.

The w-shape variation trend of the loss factor of MRE cannot depend entirely on the variation of the intrinsic damping of the rubber matrix. The interfacial friction of the particle/matrix interface is the other influencing factor. According to the bonding status, the particle/matrix interface can be divided into strong interface and weak interface [26,28] ideally. The strong interface damping is given by

(3) $$D_{fs}=\frac{2\left(1-v_{m}\right)\Phi_{p}}{\pi^{2}\left(2-v_{m}\right)}\Delta_{1}^{2}$$

where *v_m_* is the Poisson’s ratio of the rubber matrix, Φ_p_ is the volume fraction of the ferromagnetic particles, and ∆_1_ is the stress concentration factor in the slip direction. For the weak interface damping, it is given by

(4) $$D_{fw}=\frac{3\pi }{2}{f\Phi }_{p}{}_{}\Delta_{2}$$

where f is the friction coefficient between the rubber matrix and the ferromagnetic particles, and ∆_2_ is the stress concentration factor in the normal direction. Therefore, according to the mixing rate principle, the interfacial friction of MRE is given by

(5) $$D_{f}=\left(1-\varphi \right)D_{f}^{s}+{\varphi D}_{f}^{w}$$

where D_f_ is the interfacial friction damping of MRE and φ is the volume fraction of the weak bonding interface. The interfacial friction damping in the weak bonding interface is much bigger than that in the strong bonding interface. So, the interfacial friction damping increases when the volume fraction φ of the weak bonding interface increases. When exposed to gamma radiation, the bonding strength of the particle/matrix interface changes with increasing gamma radiation dose. According to the discussion in Section 3.1, the volume fraction of the strong bonding interface increases with increasing gamma radiation doses when the gamma radiation dose is lower than 8 × 10^4^ Gy(Si). When the gamma radiation dose is higher than 8 × 10^4^ Gy(Si), the volume fraction of the weak bonding interface increases with increasing gamma radiation dose. Therefore, the interfacial friction damping decreases first and then increases with increasing gamma radiation dose. It shows a V-shape variation trend with increasing gamma radiation dose. The turning point is around 8 × 10^4^ Gy(Si).

So, the damping of MRE is the combined of the intrinsic damping and the interfacial friction damping. Both the intrinsic damping and the interfacial friction damping show V-shape variation trends with increasing gamma radiation dose. But the turning points are different. For the intrinsic damping, the turning point is around 2 × 10^4^ Gy(Si). And for the interfacial friction damping, the turning point is around 8 × 10^4^ Gy(Si).The combined actions of the intrinsic damping and the interfacial friction damping result in the w-shape variation trend of the loss factor of MRE with increasing gamma radiation dose.

## 4. Conclusions

The influence of gamma radiation on the damping property of MRE was investigated by experiment in this work. The highest gamma radiation dose applied to MRE samples was up to 1 × 10^5^ Gy(Si), which can cover most of the engineering application scenarios. The effects of gamma radiation on the damping-strain relation, damping-magnetic-field relation and the variation of damping with constant strain amplitude and constant magnetic flux density were studied in detail and the probable mechanisms were discussed. It is found that the gamma radiation does not affect the variation trend of loss factor of MRE with increasing strain amplitude. But it affects the maximum strain-induced change of loss factor of MRE. The maximum strain-induced change of loss factor of MRE decreases first and then increases with increasing gamma radiation dose, which is resulted from the change of the particle/matrix interface by the gamma radiation. For the damping-magnetic-field relation, the gamma radiation does not affect the variation trend of loss factor of MRE with increasing external magnetic flux density. And the maximum magnetic-field-induced change of loss factor of MRE varies slightly due to the slight change of the magnetic property of the ferromagnetic particles with increasing gamma radiation dose. Under constant strain amplitude and constant magnetic flux density, the loss factor of MRE shows a w-shape variation trend with increasing gamma radiation dose. Both the intrinsic damping of MRE and the interfacial friction damping of MRE show V-shape variation trends with increasing gamma radiation dose. But the turning points are different. And thus, the combined actions of the intrinsic damping and the interfacial friction damping result in the w-shape variation trend of the loss factor of MRE. The study in this work would be helpful in the fabrication of MRE material and the design of MRE based device when considering the gamma radiation environment.

## Data Availability

The data presented in this study are available on request from the corresponding author.
